# Nipah Virus Disease: Recent Perspective and One Health Approach

**DOI:** 10.5334/aogh.3431

**Published:** 2021-10-12

**Authors:** Monil Singhai, Ruchi Jain, Sarika Jain, Manju Bala, Sujeet Singh, Rajeev Goyal

**Affiliations:** 1 National Centre for Disease Control, IN; 2 Regional Office Health & Family Welfare Thiruvananthapuram, IN; 3 National Tuberculosis Institute, Bangalore, IN; 4 National centre for Disease Control Delhi, IN; 5 Lady Hardinge Medical College Delhi, IN

## Abstract

**Background:**

Nipah virus (NiV) first emerged in 1998 in Malaysia, causing an outbreak of respiratory illness and encephalitis in pigs. Pig-to-human transmission of NiV associated with severe febrile encephalitis was described, and it was thought to occur through close contact with infected animals. The first outbreak was reported in India in Siliguri, West Bengal in 2001 followed by Nadia, West Bengal and adjoining areas of Bangladesh in 2007, where an intermediate animal host was not identified, suggesting bat-to-human and human-to-human transmissions. Although it is extremely difficult to document the spillover event and ascertain crossing of trans-natural boundaries by bats and bringing new viruses in an unexposed population, efforts for source identification are important to understand the epidemiology of disease. As the disease transcends beyond one species and has shown to infect humans, it therefore requires the ‘One Health approach’ in which multiple sectors coordinate and work together to achieve better public health outcomes.

**Objective:**

We summarize the re-emergence and response of the Nipah virus outbreaks (NiVD) in Kerala, India, about 1800 kms away, a decade later in 2018 and 2019. The paper recapitulates involvement of various stakeholders from the Ministry of Health and Family Welfare, Directorate of Health Research, Indian Council of Agricultural Research, State Health Department, State Animal Husbandry, District Administration, and multidisciplinary response mechanism during the NiVD outbreaks of 2018 and 2019.

**Methods:**

Information was collected from the Press Information Bureau (PIB), media/weekly alerts from the Integrated Disease Surveillance Programme (IDSP), news articles from print and electronic media, newsletters, advisories from the National Centre for Disease Control (NCDC), Disease Outbreak News (DON), World Health Organization (WHO), and published papers from various stakeholders.

**Findings & Conclusion:**

The evidence of NiV in humans and bats, with samples collected from the outbreak sites, was laboratory confirmed. The multidisciplinary response mechanisms during the 2018 outbreak helped in further understanding the importance of the One Health approach for systemic and streamlined response utilizing existing surveillance systems. This was of utmost help in the subsequent outbreak of the disease that occurred during 2019, wherein there was no documented spread of disease from the index case and no mortality was observed. This success reiterates the need for institutionalizing the involvement and cooperation of various departments and organizations during public health emergencies, especially of Zoonotic diseases, using the One Health approach.

Nipah Virus (NiV) belongs to the family paramyxovirdae genus Henipavirus with a broad host range. NiV was initially identified to be responsible for outbreaks of febrile respiratory illnesses among veterinary stock. Selected species of bats are being increasingly recognized as reservoir hosts for viruses that can cross species barriers to infect humans, other domestic, and wild mammals. NiV infection in humans causes a range of clinical presentations, from asymptomatic infection (subclinical) to acute respiratory infection and fatal encephalitis [1].

Fruit bats of the Pteropodidae family are the natural host of NiV [2]. The infected bats shed the NiV in their excretions and secretions such as feces, saliva, urine, and birthing fluids. However, bats as host reservoirs remain asymptomatic. Bats fly daily in pursuit of food, and many species fly long distances during seasonal migrations, which makes them exquisitely suitable hosts of viruses and other disease agents [3]. Human infections with NiV are rare, which suggests that the shedding of transmissible virus by bats is also a rare spillover event or occurs too infrequently to cause human infection.

We summarize the re-emergence of the Nipah virus disease (NiVD) outbreaks (2018 and 2019) in Kerala, India about 1800 kms away, a decade later from the previous ones West Bengal (2001) and adjoining areas of Bangladesh (2007). We also recapitulate the systemic and combined efforts of various stakeholders, including Central as well as State authorities, for effective and timely management of these recent outbreaks. The steps taken by all the stakeholders to mount an effective response led to successful containment of the outbreak and prevented its spread to other neighboring districts and states, limiting it to a localized event. This success also reiterates the need for involvement and cooperation of various departments and organizations during public health emergencies, especially of Zoonotic diseases and dealing with them using the One Health approach. The One Health approach aims to establish an inter-sectoral coordination mechanism at National, State, and District levels, and utilizes the existing surveillance systems of various stakeholders to detect early warning signals of impending outbreaks for timely and effective public health response; also, it facilitates sharing of relevant information within the stakeholders for taking necessary action.

## Preparedness plan and Interdisciplinary approach

After reports of an emergence of NiVD cases in Kerala in (2018 and 2019), a multi-disciplinary Central team was deputed, at the direction of the Union Health Minister, to review and respond to the situation of the NiVD in Kerala. Multi-disciplinary central teams were deployed at the sites to provide assistance to the local state authorities in investigating and responding to both the outbreaks; they consisted of public health experts, microbiologists, neurologists, and other infectious disease experts. These experts came from several organizations, including the National Centre for Disease Control (NCDC), Delhi and Calicut, Emergency Medical relief (EMR), MoHFW Delhi, All India Institute of Medical Sciences (AIIMS), Delhi, National Institute of Mental Health and Neuro-Sciences (NIMHANS), Bangalore, and National Institute of Virology Pune (NIV). Experts from the National Institute of High Security Animal Diseases (NIHSAD), Bhopal were also involved, along with the local Animal Husbandry Department [4,5,6].

**Figure 1:**
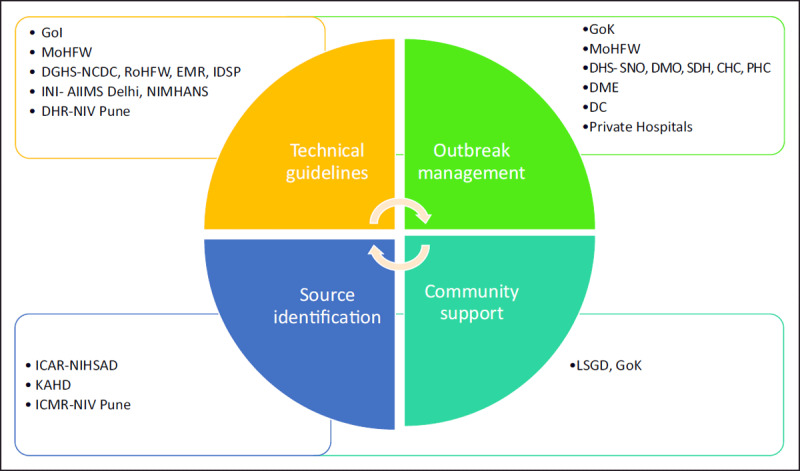
Interdisciplinary Coordination at National, State and District level. **Abbreviations**: GoI- Government of India, GoK- Government of Kerala, MoHFW- Ministry of Health & Family Welfare, DGHS- Directorate General Health Services, NCDC- National Centre for Disease Control, RoHFW- Regional office & Health Family welfare, EMR- Emergency Medical Relief, IDSP- Integrated Disease Surveillance Programme, DHS- Directorate Health Services, SNO- State Nodal Officer, DMO- District Medical Officer, SDH-Sub-district Hospital, CHC- Community Healthcare Centre, PHC- Primary Healthcare Centre, INI- Institutes of National importance, AIIMS- All india institute of medical science, NIMHANS- National Institute Of Mental Health And Neurosciences, DME- Directorate Medical Education, GMC-Government Medical College, DC- District Commissioner ICAR-Indian Council Agricultural Research, KAHD- Kerala Animal Husbandry Department, LSGD- Local Self Development Government.

As the state had a very recent experience in dealing with the NiVD outbreak, resulting in an already existing network of inter-sectoral coordination in 2018, an available set of guidelines and an expert team of doctors with past experience were already at hand for handling the outbreak of 2019 [7]. The various stakeholders coordinated a multidisciplinary response to verify the diagnosis, control the outbreaks, and identify the source of infection as depicted in ***Figure 1*** and ***Table 1***.

**Table 1:** Role of various stakeholders and coordinated multidisciplinary response mechanism l during Nipah virus disease outbreaks (2018 and 2019) at National, State, District, and Subdistrict levels.


CENTRAL AUTHORITIES		

PARTNER INSTITUTES	EXPERTISE	ROLES & RESPONSIBILITIES

1. NCDC Delhi & NCDC Kozhikode 2. ROHFW Thiruvananthapuram 3. EMR, 4. AIIMS Delhi 5. NIMHANS Bangalore 6. NIV Pune	Public health experts Neurologists Infectious disease experts Microbiologists	1. Providing technical guidance on Case Definitions for Suspect, Probable, Confirmed cases and Contacts, advisory for healthcare personnel, Information for general public, Treatment guidelines, Sample collection and transport guidelines 2. Establishing the timelines of events, undertaking epidemiological linkage and risk assessment 3. Verify the outbreak and confirm the diagnosis 4. Suggest measures for containment 5. Training for ICP, including Dead body disposal 6. Establishing point of care test (PoCT) during the second outbreak 7. Activation of Emergency Operations Centre (EoC) 8. Undertake reporting of event to International Agencies, as prescribed under IHR

1. NIHSAD Bhopal	Veterinary expert	1. Source identification collection of bats and testing for NiVD

**STATE, DISTRICT AND SUBDISTRICT AUTHORITIES**		

1. Health and Family Welfare Department, Government of Kerala including Govt Medical Colleges Kozhikode, Ernakulam, Thrissur and District Hospitals of Thrissur and Idukki 2. Private hospitals 3. Municipal Corporation	Directorate of Health Services, Kerala State Programme Officer, IDSP Public health experts Microbiologist Clinicians Other Healthcare Workers	1. Strengthening Infection control practices and Biomedical waste management practices 2. Early suspicion of cases 3. Establishing Control rooms and providing resources for response 4. Treatment of cases and Isolation of suspects 6. Cremation and last rites of the deceased as per the guidelines 7. Contact tracing and establishing epidemiological linkages 8. Undertake Media briefings

2. Kerala Animal Husbandry Department	Veterinary expert	1. Source identification and collection of bats 2. Establishing and attending helpline for livestock farmers 3. Vigil on reports of livestock for unusual symptoms

4. Nipah cell (District Rapid Response Team)	Public health experts IT cell	1. Initiating the first response to the outbreak 2. Attending helpline, screening calls, follow up of symptomatic cases through call back 3. Cyber space monitoring 4. Social media awareness through IEC, posters and videos

5. District Administrative authorities	District Commissioner District Police	1. Cyber Investigation for online fake content related to NiVD 2. Maintain law and order

6. Local Self Government Department(LSGD) (refr) comprising Panchayat Directorate, Directorate of Urban Affairs, Commissionerate of Rural Development and Town and Country Planning Department	Representatives of local bodies of the district	1. Co-ordinate the above activities and provide resource assistance 2. Help in alleviating the fears of community 3. Provide support to affected families and food kits to home quarantine cases 4. Facilitating implementation of surveillance and response


## Outbreak description

The first outbreak of NiVD was reported in India in District Siliguri, West Bengal in 2001 (66 probable cases and 45 deaths) followed by an outbreak in District Nadia, West Bengal and adjoining areas of Bangladesh in 2007 (5 probable cases and 5 deaths). As any intermediate animal host was not identified, bat-to-human and human-to-human transmissions were suggested [8,9].

### 2018

The unusual event apparently started with the death of a 26-year-old male resident of Block Perambra at the PHC-Changaroth on 5 May 2018, who was clinically diagnosed with viral encephalitis at the Government Medical College Hospital, Kozhikode on 4 May 2018. Two weeks later, two members of the same family reported with similar symptoms, deteriorated rapidly and died while in treatment at a private tertiary care hospital. Then, a cluster of three cases of viral encephalitis was reported from the same area. Subsequently, six more persons succumbed to similar symptoms, including a staff nurse of the taluk hospital involved in treatment of the primary/index case. All the affected cases were found to be epidemiologically linked with a confirmed case/death. Laboratory testing of throat swabs, urine, and blood samples collected from four suspected patients was conducted by the National Institute of Virology, Pune; three of the four reported deaths were confirmed positive for NiV by real-time polymerase chain reaction (RT-PCR) and IgM Elisa for NiV, and thereafter many more. A total of 19 cases and 17 deaths were reported through 3 June 2018 [10,11,12]. The State authorities identified a total of 2,084 contacts of the 19 cases from the village panchayats, who were under surveillance, and none of them developed NiVD [13]. The State Government declared the affected areas NiVD free on 1 July 2018 [14].

### 2019

On 30 May 2019, a 23-year-old male resident of Vadakkekara Village, Paravur Block, Ernakulam District, Kerala, having fever with altered sensorium, was referred to a private hospital at Eranakulam. A throat swab, CSF, blood, and urine samples were collected. The samples tested positive and were confirmed for NiV (Real-time RT-PCR, nested RT-PCR, and for anti-Nipah human IgM and IgG antibodies by ELISA assay) by NIV Pune. A total of 330 contacts were traced and samples from all the 13 suspected cases were tested and found negative for NiV [15,16]. Ernakulam, Thrissur, and Idukki districts were put under surveillance for high index of exposure to NiVD. The young patient recovered and no other cases were reported subsequently, hence it was considered a localized event [17].

## Implementing and monitoring of strategies to detect and prevent cases

For early detection, monitoring, and response to epidemic-prone disease outbreaks (including NiVD), the Government of India is doing surveillance through the Integrated Disease Surveillance Program (IDSP) and providing technical and financial support to all states under the National Health Mission (NHM). The NiVD guidelines for case definitions (for suspected, probable, and confirmed cases) and contacts, advisories for healthcare personnel, information for the general public, treatment guidelines, sample collection, and transport guidelines were framed by IDSP and NCDC [18,19].

The District Rapid Response Teams, along with the central multidisciplinary team, investigated the outbreaks in 2018 and 2019. House to house active case searching and contact tracing was done. Nipah cell of the state was established for contact tracing and ensuring home quarantine. Control rooms were set up at the State Headquarters and District Headquarters. Core committee daily review meetings were held under the chairmanship of the State Health Minister and District Collectors. Central teams reviewed the treatment procedure, infection control practices, use and availability of personal protective equipment (PPE), and availability of drugs, particularly in the designated hospitals [20]. An action plan was also developed for the healthcare staff and a simulation exercise was planned. Risk communication messages were delivered to the community, public, stakeholders, and partners. Training was organized for infection control practices, specimen transportation, dead body handling, etc [4,5,6].

## Outbreak Management at Hospital & Community level

Standard contact, droplet, and airborne precautions were emphasized during patient care and aerosol generating procedures to prevent human to human transmission while managing probable/suspected or confirmed cases of NiVD [21]. To control the outbreak in hospital settings, dedicated isolation areas were identified, and infection control protocols were strengthened. Training sessions for HCW in infection control and treatment regarding NiV were organized [22]. Protocols for transportation and burial of deceased bodies were issued [23,24]. Home quarantine for the contacts, hospital-based monitoring for all symptomatic persons, and specific treatment guidelines were implemented [20]. Medical camps were conducted in the area for providing food kits to home quarantine cases, creating disease awareness and reducing panic in the community through the Local Self Government Department, Kerala [25]. Awareness programs were carried out through electronic and print media so as to allay fear in the community and to prevent the spread of rumors [26].

## Technical challenges faced and interventions done in response

### Surveillance strengthening

The NiVD outbreak in 2018 was a major event with a very high case fatality rate (CFR), as characteristic of NiVD; however, the subsequent outbreak can be termed as a minor event. The primary cases in both outbreaks could have been infected by an accidental spillover from NiV infected bats or food sources contaminated by their secretions. However, in the 2018 outbreak, the initial spillover event may have been amplified by person-to-person transmission in the hospital settings, as subsequent cases were either close family contacts or hospital contacts. These two episodes are likely to be isolated/localized incidents.

The unexpected sudden death of an otherwise healthy young male during the 2018 outbreak could have generated early warning signals only if such unusual events were reported diligently. As all the cases were epidemiologically linked to the index case, (family contact, healthcare facility contacts), subsequent infection/mortality could have been prevented if the infection control practices and BMW management practices at various levels of healthcare centers (Primary Healthcare, Community Healthcare, and Hospitals) were stringently followed. Thus, adequate quantities of PPE and other logistics were ensured in the district at the earliest. Standard precautions and safe BMW practices were also emphasized while handling the deceased, patient specimens, used PPE, linen, clothing of patients, cleaning, and waste disposal activities at all levels of healthcare centers.

Lack of an upgraded virus testing laboratory in the state, especially for dealing with BSL-3 or 4 pathogens, poses an additional burden of sample transportation with biocontainment precautions and delayed response during such outbreak situations [27]. The need was felt during the 2018 outbreak for setting up of regional/state-level advanced testing/point-of-care testing (PoCT) locations that are equipped with biocontainment facilities for dealing with these pathogens. A PoCT for NiVD was established by the NIV Pune at the Government Medical College Ernakulam during 2019 [28].

### Source identification

Pig-to-human transmission of NiV associated with severe febrile encephalitis has been described in past NiVD outbreaks [29]. Human-to-human transmission was established during the outbreak of 2018, but the mystery of the source of infection was yet to be solved. The teams from ICAR, ICMR, and the State Animal Husbandry and Forest departments put a concerted effort to solve this mystery. It was suggested to enhance surveillance in livestock and to examine bats, which are natural reservoirs of NiV. Ten out of 55 *Pteropus giganteus* bats were found positive for NiV by RT-PCR [30].

During the 2019 outbreak, one rectal swab sample (out of 141 swab samples of bats) and 3 bat visceral organs (out of 92 bats) were found positive for NiV. Interestingly, 20.68% (12/58) of *Pteropus sp* were positive for anti-NiV IgG antibodies. As per genetic analysis of NiV conducted by ICMR- NIV (Pune) and NIV (Allaphuza, Kerala), a distinct cluster of NiV sequences suggested the circulation of a new genotype (I-India) in South India, which is different from the Bangladesh and Indian northeast NiV strains [31]. No clinical manifestation of NiV was seen among the livestock population in the vicinity. All efforts were made to ensure that the infection was not transmitted to animals from humans through excreta, leftover food, clothes, and the like.

### Fear Psychosis & Misleading Media

In absence of a clearly identified primary source of infection to the index/primary case, many unscientific and misleading reports (especially through social media) on the possible transmission of disease from fruits, such as mangoes, lychees, dates, bananas, etc., created fear and trepidation in the community [32]. Due to fear generated by these rumors, people started vacating their homes in the affected Panchayats. NiVD was even considered a conspiracy of the global drug ‘mafia’ or an ‘elaborate hoax’ by some pharmaceutical companies to boost their pharmaceutical products [33]. A public meeting was arranged by the local body to dispel the doubts of the locals and prevent fear and rumor-mongering through social media. The media was made a partner in disseminating health messages. Transparent and controlled communications were maintained with the media and public [34]. Erring social media posts were scrutinized and penalized by the administration [33]. As even close relatives were staying away from the deceased due to the fear of contracting the deadly virus, the state health department took the responsibility of cremation and last rites of the deceased as per the advisories [24].

The establishment of strong surveillance and multidisciplinary response mechanisms during the 2018 outbreak was of utmost help in mounting a systematic and organised response rapidly and effectively during the 2019 outbreak, preventing significant human transmission and mortality.

## Lessons learned post intervention for future activities

### Strengthening the healthcare system

The clinical care of patients with suspected or confirmed highly pathogenic infectious diseases is a real challenge for healthcare facilities [35]. A separate outpatient facility for fever patients should be set up at all hospitals in the district. Standard operating protocols for infection control and BMW management practices should be implemented at every level of healthcare facility and monitored regularly. Well-equipped isolation wards (adequate infection control practices in place) for patients with symptoms suggestive of highly infectious/contagious diseases, such as NiVD or Ebola, are essential to reduce the risk of the spread of nosocomial infection in hospital settings. The state must ensure that suspect cases of highly pathogenic infectious diseases do not go to routine referral healthcare systems but must be managed at designated tertiary care facilities with appropriate infection control and biomedical waste management practices.

The conventional clinical diagnostic procedure required for confirmation of highly infectious disease requires advanced biocontainment laboratories, high-end (and costly) instruments, an expert technician for operation and result interpretation, and longer turnaround time. The technical advancements in research and translation to development of PoCT facilities must be encouraged, which will elevate the clinical diagnostic scenario in resource-limited settings.

### Public-Private Partnership

As per the ensuing bat breeding season, NiVD advisories must be issued to clinicians in affected areas for high index of suspicion and appropriate infection control practices both in public and private sectors [36]. The timely suspicion of cases with unusual illness at private hospital settings and follow-up during both the outbreaks was the first clue that put in place the spree of coordinated events from district through national levels. The success stories of strengthened public-private partnership to achieve health for all in various parts of the world warrants a call for attention on this aspect in our country. As patients with unusual illness can also present at private healthcare facilities, there should be a clear channel defined for referral and reporting. Physicians in private practices should also be routinely sensitized and trained in identification and reporting for such diseases.

### IEC activities

Print and electronic media, social activists, community leaders, and nongovernment organizations play an active role in early case reporting, social mobilization, and the raising of public awareness, which is essential in the rapid control of any outbreak. To prevent the spread of false information, officers must be designated to give authentic information to the media. Correct information helps in managing panic amongst the general public, which can otherwise affect the containment efforts of authorities. A grassroots approach to public health was exercised with success in the NiVD outbreak through consistent public health messages delivered at the community level and steered at the state level, with strategic co-ordination by central government and collaboration with local government [37].

### Inter-sectoral Coordination

The district, subdistrict, and block-level administrations, along with elected people’s representatives, should be involved in discussions not only during outbreaks, but also prior to the season, to highlight the possibility of an outbreak from endemic- and epidemic-prone diseases. Possible prevention measures can be discussed at routine intersectoral monthly meetings chaired by district or sub-district administrators, leading to better coordination and cooperation at the community level [38].

### Modelling transmission dynamics

As spring is the probable breeding season for bats and the preliminary observations with regards to time of occurrence of both events is May–June, this indicates a higher probability of the presence of the virus among bats, and therefore a spillover, during breeding season [2]. Further studies on the transmission dynamics and ecological drivers of these viruses in reservoir hosts and spillover events, with regards to seasonality, will help us to generate evidence helping surveillance systems to be on higher alert and preparedness for a coordinated public health response [17].

## Conclusion

When NiV infections are suspected, standard contact and droplet precautions and infection control practices must be promptly strengthened to avoid spillovers in healthcare facility settings, both public and private. Further, in view of emerging NiV infection in the last decades in newer areas, countrywide strengthening of existing disease surveillance systems and multidisciplinary response mechanisms is necessary to enable rapid detection of rare zoonotic diseases, such as NiVD, as is the institution of appropriate control measures with One Health strategy.
